# Ramanujan’s partition generating functions modulo $\ell$

**DOI:** 10.1007/s11139-025-01241-0

**Published:** 2025-11-04

**Authors:** Kathrin Bringmann, William Craig, Ken Ono

**Affiliations:** 1 Department of Mathematics and Computer Science, Division of Mathematics, University of Cologne, Weyertal 86-90, 50931 Cologne, Germany; 2 Department of Mathematics, United States Naval Academy, 572C Holloway Road Mail Stop 9E, Annapolis, MD 21402 USA; 3 Department of Mathematics, University of Virginia, Charlottesville, 22904 USA

**Keywords:** Modular forms, Partition function, Ramanujan’s partition congruences, 05A17, 11P82

## Abstract

For the partition function *p*(*n*), Ramanujan proved the striking identities

$$\begin{aligned} \begin{aligned} \mathcal {P}_5(q):=\sum _{n\ge 0} p(5n+4)q^n&=5\prod _{n\ge 1} \frac{\left( q^5;q^5\right) _{\infty }^5}{(q;q)_{\infty }^6},\\ \mathcal {P}_{7}(q):=\sum _{n\ge 0} p(7n+5)q^n&=7\prod _{n\ge 1}\frac{\left( q^7;q^7\right) _{\infty }^3}{(q;q)_{\infty }^4}+49q \prod _{n\ge 1}\frac{\left( q^7;q^7\right) _{\infty }^7}{(q;q)_{\infty }^8}, \end{aligned} \end{aligned}$$

where $(q;q)_{\infty }:=\prod _{n\ge 1}(1-q^n).$ As these identities imply his celebrated congruences modulo 5 and 7, it is natural to seek, for primes $\ell \ge 5,$ closed form expressions of the power series

$$ \mathcal {P}_{\ell }(q):=\sum _{n\ge 0} p(\ell n-\delta _{\ell })q^n\pmod {\ell }, $$

where $\delta _{\ell }:=\frac{\ell ^2-1}{24}.$ In this paper, we prove that

$$ \mathcal {P}_{\ell }(q)\equiv c_{\ell } \dfrac{\mathcal {T}_{\ell }(q)}{\left( q^\ell ; q^\ell \right) _\infty } \pmod {\ell }, $$

where $c_{\ell }\in \mathbb Z$ is explicit and ${\mathcal {T}}_{\ell }(q)$ is the generating function for the Hecke traces of $\ell$-ramified values of special Dirichlet series for weight $\ell -1$ cusp forms on $\textrm{SL}_2(\mathbb Z)$. This is a new proof of Ramanujan’s congruences modulo 5, 7, and 11, as there are no nontrivial cusp forms of weight 4, 6, and 10.

## Introduction and statement of results

A *partition* of *n* is any nonincreasing sequence of positive integers that sum to *n*. The number of partitions of *n* is denoted *p*(*n*) (by convention, we let $p(0):=1$ and $p(n):=0$ for $n<0$). Ramanujan famously proved (see [2, 7]), for every non-negative integer *n*, that

$$\begin{aligned} \begin{aligned} p(5n+4)&\equiv 0\pmod 5,\\ p(7n+5)&\equiv 0\pmod 7,\\ p(11n+6)&\equiv 0\pmod {11}. \end{aligned} \end{aligned}$$

For the congruences with modulus 5 and 7, he used the beautiful identities

$$\begin{aligned} \begin{aligned} \mathcal {P}_5(q):=\sum _{n\ge 0} p(5n+4)q^n&=5\prod _{n\ge 1} \frac{\left( q^5;q^5\right) _{\infty }^5}{(q;q)_{\infty }^6},\\ \mathcal {P}_{7}(q):=\sum _{n\ge 0} p(7n+5)q^n&=7\prod _{n\ge 1}\frac{\left( q^7;q^7\right) _{\infty }^3}{(q;q)_{\infty }^4}+49q \prod _{n\ge 1}\frac{\left( q^7;q^7\right) _{\infty }^7}{(q;q)_{\infty }^8}, \end{aligned} \end{aligned}$$

where $(q;q)_{\infty }:=\prod _{n\ge 1}(1-q^n).$ In 1969, with the help of binary theta functions, Winquist [8] was able to offer another identity that proved Ramanujan’s congruence with modulus 11.

In the spirit of these identities, for every prime $\ell \ge 5,$ we determine the *q*-series $\mathcal {P}_{\ell }(q)\in \mathbb {F}_{\ell }[[q]]$

$$\begin{aligned} \mathcal {P}_{\ell }(q):=\sum _{n\ge 0} p(\ell n-\delta _{\ell })q^n\pmod {\ell }, \end{aligned}$$

where $\delta _{\ell }:=\frac{\ell ^2-1}{24}.$ These expressions involve the generating functions of “weighted Hecke traces” of special values of specific Dirichlet series associated to weight $\ell -1$ Hecke eigenforms on $\textrm{SL}_2(\mathbb Z)$ (for background see [3] or [6]).

To define these Hecke traces, first suppose that ($q:=e^{2\pi i z}$ throughout)

$$f(z):=q+\sum \limits _{n\ge 2}a_f(n)q^n\in S_{2k}$$

is an even integer weight 2*k* Hecke eigenform on $\textrm{SL}_2(\mathbb Z)$. For $s\in \mathbb {C}$ with $\Re (s)>2k,$ the *twisted quadratic Dirichlet series* is defined by

$$\begin{aligned} D(f;s):=\sum \limits _{n\ge 1}\frac{\left( \frac{12}{n}\right) a_f\left( \frac{n^2-1}{24}\right) }{n^s}, \end{aligned}$$

where $\left( \frac{\cdot }{\cdot }\right)$ denotes the Kronecker symbol. Note that we set $a_f(n):= 0$ if $n\notin \mathbb Z$. Furthermore, if $k\ge 2$, $0\le j\le k-2$, and $m\ge 0,$ then we let

$$\begin{aligned}&\beta (k,j,m):=\frac{(-1)^{j+1}\Gamma \!\left( k-\frac{1}{2}\right) \Gamma \!\left( k+\frac{1}{2}\right) }{9}\left( \frac{6}{\pi }\right) ^{2k}\\&\quad \frac{(2k+m-2)!(k-j-1)^{[k]}\left( \frac{3}{2}\right) ^{\![j]}}{j! m! (2k-j-2)!\left( -\frac{1}{2}-j\right) ^{\![k]}\left( \frac{5}{2}\right) ^{\![j]}}, \end{aligned}$$

where $\Gamma (\cdot )$ is the usual Gamma-function. Moreover the *rising factorial* is given by

$$\begin{aligned} (x)^{[j]}:={\left\{ \begin{array}{ll} x(x+1)\cdots (x+j-1) \ \ \ \ \   & {\text {if}}\ j\ge 1,\\ 1 \ \ \ \ \   & {\text {if } j=0}, \end{array}\right. } \end{aligned}$$

which are companions of the usual *falling factorials*

$$\begin{aligned} (x)_m:={\left\{ \begin{array}{ll} x(x-1)\cdots (x-m+1) \ \ \ \   & {\text {if } m\ge 1},\\ 1 \ \ \ \ \   & {\text {if } m=0},\\ \frac{1}{(x)_{-m}} \ \ \ \ \   & {\text {if } m\le -1}. \end{array}\right. } \end{aligned}$$

For such $f\in S_{2k},$ we define the following sums of values of Dirichlet series by^1^

$$\begin{aligned} D_f:= \sum \limits _{j=0}^{k-2}\sum _{m\ge 0} \beta (k,j,m) D(f;2k+1+2m+2j). \end{aligned}$$

Moreover we define, for $n\in \mathbb {N}$, the *weight 2k Hecke trace* by

$$\begin{aligned} {{\,\textrm{Tr}\,}}_{2k}(n):=\sum _{f} a_f(n) \frac{D_f}{||f||}, \end{aligned}$$

where the sum runs over the normalized Hecke eigenforms $f\in S_{2k},$ and the Petersson norms of *f*, ||*f*||, is defined as ($z=x+iy$ throughout)

$$ ||f||:=\int _{\textrm{SL}_2(\mathbb Z)\backslash \mathbb {H}} |f(z)|^2 y^{2k}\frac{dxdy}{y^2}. $$

As $a_f(n)$ is the eigenvalue of the Hecke operator $T_n$, we refer to the numbers ${{\,\textrm{Tr}\,}}_{2k}(n)$ as Hecke traces. Finally, for primes $\ell \ge 5,$ we collect the $\ell$-ramified values (i.e., the arguments that are multiples of $\ell$) if $2k=\ell -1$ as the Fourier coefficients of the generating function

$$\begin{aligned} \mathcal {T}_{\ell }(q):=\sum _{n\ge 1} {{\,\textrm{Tr}\,}}_{\ell -1}(\ell n)q^n. \end{aligned}$$

### Theorem 1.1

If $\ell \ge 5$ is a prime, then

$$ \mathcal {P}_{\ell }(q)\equiv c_{\ell } \dfrac{\mathcal T_{\ell }(q)}{\left( q^\ell ; q^\ell \right) _\infty } \pmod {\ell }, $$

where $c_{\ell }:= 2\cdot \overline{3} (\frac{-1}{\ell }) (\frac{\ell +1}{2})!^{\ell -3} \pmod {\ell }$, where throughout ${\overline{a}}$ denotes the inverse of $a\pmod {\ell }$ and where $(\frac{\cdot }{\cdot })$ denotes the Kronecker symbol.

For $\ell \in \{5, 7, 11\}$, we have that $S_{\ell -1}=\{0\}.$ As there are no nontrivial cusp forms in these spaces, we immediately obtain a new proof of Ramanujan’s famous partition congruences.

### Corollary 1.2

For $n\in \mathbb {N}$, we have

$$\begin{aligned} \begin{aligned} p(5n+4)&\equiv 0\pmod 5,\\ p(7n+5)&\equiv 0\pmod 7,\\ p(11n+6)&\equiv 0\pmod {11}. \end{aligned} \end{aligned}$$

Moreover Theorem 1.1 immediately implies the following congruence formula for $p(\ell n-\delta _{\ell })\pmod \ell$ in terms of $p(0), p(1),\dots , p(n-1)$.

### Corollary 1.3

If $\ell \ge 5$ is a prime and $n\in \mathbb {N}$, then we have

$$ p(\ell n-\delta _{\ell }) \equiv c_{\ell } \sum _{\begin{array}{c} j,m \ge 0 \\ \ell j+m=n \end{array}} p(j) {{\,\textrm{Tr}\,}}_{\ell -1}(\ell m)\pmod \ell . $$

### Example

For the prime $\ell =13,$ Theorem 1.4 and Corollary 1.3 of [4] gives

$$\begin{aligned} \mathcal {T}_{13}(q)&= -\dfrac{33108590592}{691} \Delta |U_{13}(z) \equiv 7 \Delta |U_{13}(z) \pmod {13}, \end{aligned}$$

where $f|U_j(z):=\sum _{n\ge 1} a_f(jn)q^n$ for $j\in \mathbb {N}$. Using $c_{13} \equiv 6 \pmod {13}$, we obtain

$$\begin{aligned} \begin{aligned}&c_{13} \dfrac{\mathcal T_{13}(q)}{\left( q^{13}; q^{13} \right) _\infty } \equiv \dfrac{3\Delta |U_{13}(z)}{\left( q^{13}; q^{13} \right) _\infty }\\&\quad \equiv 11q+9q^2+3q^3+6q^4+12q^5+6q^6+q^8+\dots \pmod {13}. \end{aligned} \end{aligned}$$

To illustrate Theorem 1.1, we note that

$$\begin{aligned} \begin{aligned} \mathcal {P}_{13}(q)\!&=\!\sum _{n \ge 1} p(13n\!-\!7)q^n\!=\!11q\!+\!490q^2\!+\!8349q^3\!+\!89134q^4\!+\!715220q^5\!+\!\dots \\&\equiv 11q+9q^2+3q^3+6q^4+12q^5+6q^6+q^8+\dots \pmod {13}. \end{aligned} \end{aligned}$$

Furthermore, Corollary 1.3 implies, for $n\in \mathbb {N}$, that

$$\begin{aligned} \begin{aligned} p(13n-7)&\equiv 3 \sum _{\begin{array}{c} j,m \ge 0 \\ 13j+m=n \end{array}} p(j) \tau (13m)\pmod {13}.\\ \end{aligned} \end{aligned}$$

To obtain Theorem 1.1, we make use of recent work of Gomez, the third author, Saad, and Singh [4] that offers an infinite family of generalizations of Euler’s “Pentagonal Number” recurrence for *p*(*n*). In Sect. 2 we recall these formulas, and in Sect. 3 we use them to obtain Theorem 1.1.

## Generalizations of Euler’s “Pentagonal number” recurrence

For $n\in \mathbb {N},$ Euler’s famous recurrence relation asserts that (see p. 12 of [1])

2.1 $$\begin{aligned}&p(n)=p(n-1)+p(n-2)-p(n-5)-p(n-7)+ \dots \nonumber \\&\quad = \sum _{m\in \mathbb Z\setminus \{0\}} (-1)^{m+1}p(n-\omega (m)), \end{aligned}$$

where $\omega (m):=\frac{3m^2+m}{2}$ is the *m*-th *pentagonal number*. This recurrence is one of the most efficient methods for computing partition numbers.

Gomez, the third author, Saad, and Singh [4] proved that Euler’s recurrence is the first case of an infinite family of rich recurrence relations satisfied by the partition numbers. To make this precise, we make use of *Dedekind’s eta-function*

$$\begin{aligned} \eta (z):=q^{\frac{1}{24}}\prod _{n\ge 1}\left( 1-q^n\right) =\sum _{n\in \mathbb Z} (-1)^n q^{\frac{1}{24}(6n+1)^2}, \end{aligned}$$

where $z\in \mathbb {H},$ the upper half of the complex plane. To define these relations, we require the differential operator $D:=\frac{1}{2\pi i}\frac{d}{dz} = q\frac{d}{dq}.$ For $k\in \mathbb {N}_0,$ we define^2^,^3^

$$\begin{aligned} R_{k}(z):= \frac{(2k-1)(2k-2)_{k-1}^2}{2^{2k-2}} \sum _{\begin{array}{c} r,s \ge 0 \\ r + s = k \end{array}} (-1)^{r+1} \frac{2s-1}{(2r)! (2s)!} D^r\left( \frac{1}{\eta (z)}\right) D^s(\eta (z)). \end{aligned}$$

By [4], we have

$$\begin{aligned} R_{k}(z)= \sum _{\begin{array}{c} n\ge 0\\ m\in \mathbb Z \end{array}} (-1)^{m+1} g_{k}(n,m) p(n-\omega (m))q^n, \end{aligned}$$

where

$$\begin{aligned} g_k(n,m):=\frac{(2k-1)(2k-2)_{k-1}^2}{2^{2k-2}}  &   \sum _{r=0}^{k} (-1)^{k+r}\frac{2k-2r-1}{ (2r)! (2k-2r)!} (6m+1)^{2r}\\  &   \quad \left( 24n - (6m+1)^2\right) ^{k - r}. \end{aligned}$$

By Theorem 1.1 of [4], for each $k \ge 0$, $R_{k}$ is a weight 2*k* holomorphic modular form on $\textrm{SL}_2(\mathbb Z)$. These expressions are simple to compute for $k \le 13$ apart from $k=12.$ Namely, Corollaries 1.2 and 1.3 of [4] give the following identities in terms of the usual Eisenstein series

$$\begin{aligned} E_{2k}(z):= 1 - \dfrac{4k}{B_{2k}} \sum _{n \ge 1} \sigma _{2k-1}(n) q^n, \end{aligned}$$

where $B_r$ denotes the *r*-th Bernoulli number, $\sigma _r(n):=\sum _{d\mid n} d^r$ the *r-th divisor sum*, and $\Delta (z):=\eta ^{24}(z)$.

### Theorem 2.1

The following are true:

1. If $k\in \{0, 1\},$ then we have
  $$ R_{k}(z) = {\left\{ \begin{array}{ll} -1 \ \ \ \ \   & {\text {if }} k=0,\\ 0 \ \ \ \ \   & {\text {if }} k=1. \end{array}\right. } $$
2. If $k \in \{2, 3, 4, 5, 7\},$ then we have
  $$ R_{k}(z) = \left( {\begin{array}{c}2k-2\\ k-2\end{array}}\right) E_{2k}(z). $$
3. If $k \in \{6, 8, 9, 10, 11, 13\},$ then we have
  $$ R_{k}(z) = \left( {\begin{array}{c}2k-2\\ k-2\end{array}}\right) E_{2k}(z) + \beta _{k}\Delta _{2k}(z), $$
  where
  $$\begin{aligned} \Delta _{2k}(z):=q+\sum _{n\ge 2} \tau _{2k}(n)q^n:= {\left\{ \begin{array}{ll} \Delta (z) \ \ \ \ \   & {\text {if } k=6,}\\ \Delta (z)E_4(z) \ \ \ \   & {\text {if } k=8},\\ \Delta (z)E_6(z) \ \ \ \   & {\text {if } k=9,}\\ \Delta (z)E_4^2(z) \ \ \ \ \   & {\text {if} k=10,}\\ \Delta (z)E_4(z)E_6(z) \ \ \ \ \   & {\text {if } k=11,}\\ \Delta (z)E_4^2(z) E_6(z)\ \ \ \ \   & {\text {if } k=13,} \end{array}\right. } \end{aligned}$$
  where we let
  $$\begin{aligned} \beta _{k}:= {\left\{ \begin{array}{ll} -\frac{33108590592}{691} \ \ \ \ \   & {\text {if } k=6,}\\ \ \\ -\frac{187167592415232}{3617} \ \ \ \ \   & {\text {if } k=8,}\\ \ \\ -\frac{28682634201661440}{43867} \ \ \ \ \   & {\text {if } k=9,}\\ \ \\ -\frac{8294726176465158144}{174611} \ \ \ \ \   & {\text {if } k=10,}\\ \ \\ -\frac{101475065073734516736}{77683} \ \ \ \ \   & {\text {if} k=11,}\\ \ \\ -\frac{1195065734266339700244480}{657931} \ \ \ \ \   & {\text {if } k=13.} \end{array}\right. } \end{aligned}$$

Finally, for general *k*,  Theorem 1.4 of [4] gives the following expressions that make use of the weighted Hecke trace generating function

$$\begin{aligned} {T}_{2k}(z):=\sum _{n\ge 1} {{\,\textrm{Tr}\,}}_{2k}(n)q^n\in S_{2k}. \end{aligned}$$

### Theorem 2.2

If $k \ge 6,$, $k\ne 7$, then we have

$$ R_{k}(z)= \left( {\begin{array}{c}2k-2\\ k-2\end{array}}\right) E_{2k}(z) + {T}_{2k}(z). $$

These results are equivalent to the infinite family of recurrence relations given in the following corollary.

### Corollary 2.3

If *n* is a positive integer, then the following are true:

1. We have that
  $$\begin{aligned} p(n) = \sum _{m \in \mathbb Z\backslash \{0\}} (-1)^{m+1} p\left( n - \omega (m) \right) . \end{aligned}$$
2. If $k \in \{2, 3, 4, 5, 7\}$, then we have
  $$\begin{aligned}  &   p(n) =\frac{1}{g_{k}(n,0)}\\  &   \quad \left( -\frac{4k}{B_{2k}} \left( {\begin{array}{c}2k - 2\\ k - 2\end{array}}\right) \sigma _{2k - 1}(n) + \sum _{m \in \mathbb Z\setminus \{0\}} (-1)^{m+1} g_k(n,m)\,p(n - \omega (m))\right) . \end{aligned}$$
3. If $k \in \{ 6, 8, 9, 10, 11, 13 \}$, then we have
  $$\begin{aligned}  &   \hspace{.5cm}p(n) =\frac{1}{g_{k}(n,0)}\left( -\frac{4k}{B_{2k}} \left( {\begin{array}{c}2k - 2\\ k - 2\end{array}}\right) \sigma _{2k - 1}(n) +\beta _{k}\tau _{2k}(n)\right. \\  &   \quad \left. + \sum _{m \in \mathbb Z\setminus \{0\}} (-1)^{m+1} g_k(n,m)\,p(n - \omega (m))\right) . \end{aligned}$$
4. If $k\ge 2$, with $k\ne 7$, then we have
  $$\begin{aligned}  &   \hspace{.5cm}p(n) = \dfrac{1}{g_k\left( n, 0 \right) } \left( -\dfrac{4k}{B_{2k}} \left( {\begin{array}{c}2k-2\\ k-2\end{array}}\right) \sigma _{2k-1}(n) + \textrm{Tr}_{2k}(n) \right. \\  &   \quad \left. + \sum _{m \in \mathbb Z\backslash \{0\}} \left( -1\right) ^{m+1} g_k\left( n,m \right) p\left( n - \omega (m) \right) \right) . \end{aligned}$$

## Proof of Theorem 1.1

The proof of Theorem 1.1 requires the following elementary proposition regarding the congruence properties of certain examples of Corollary 2.3 (4). Namely, we obtain a pentagonal number recurrence modulo $\ell$ for the Hecke traces with argument $\ell n$, where the pentagonal numbers $\omega (m)$ are restricted to a fixed congruence class modulo $\ell$.

### Proposition 3.1

If $\ell \ge 5$ is prime and *n* is a positive integer, then

$$\begin{aligned} \textrm{Tr}_{\ell -1}\left( \ell n \right) \equiv -3\cdot \overline{2} \left( \frac{\ell +1}{2}\right) !^2 \sum _{\begin{array}{c} m \in \mathbb Z\\ 6m \equiv -1 \pmod {\ell } \end{array}} (-1)^{m+1} p\left( \ell n - \omega (m) \right) \pmod {\ell }. \end{aligned}$$

### Proof

By Corollary 2.3 (4), we have, for $k \ge 2$, that

$$\begin{aligned}  &   p(n) = \dfrac{1}{g_k\left( n, 0 \right) } \left( -\dfrac{4k}{B_{2k}} \left( {\begin{array}{c}2k-2\\ k-2\end{array}}\right) \sigma _{2k-1}(n) + \textrm{Tr}_{2k}(n) \right. \\  &   \quad \left. + \sum _{m \in \mathbb Z\backslash \{0\}} \left( -1\right) ^{m+1} g_k\left( n,m \right) p\left( n - \omega (m) \right) \right) . \end{aligned}$$

By letting $k = \frac{\ell -1}{2}$, the von Stadt–Clausen Theorem (for example, see [5, Theorem 3, p. 233]) implies that the denominator of the Bernoulli number $B_{\ell -1}$ is divisible by $\ell$, which in turn implies that the divisor function contribution above vanishing modulo $\ell$. By then letting $n \mapsto \ell n$, we obtain

3.1 $$\begin{aligned}  &   p\left( \ell n \right) \equiv \dfrac{1}{g_{\frac{\ell -1}{2}}\left( \ell n, 0 \right) } \Bigg (\textrm{Tr}_{\ell -1}\left( \ell n \right) + \sum _{m \in \mathbb Z\backslash \{0\}} \left( -1 \right) ^{m+1} \nonumber \\  &   \quad g_{\frac{\ell -1}{2}}\left( \ell n, m \right) p\left( \ell n - \omega (m) \right) \Bigg ) \pmod {\ell }. \end{aligned}$$

By direct calculation, we have

3.2 $$\begin{aligned}&g_{\frac{\ell -1}{2}}\left( \ell n, m \right) = \dfrac{\left( \ell -2 \right) \left( \ell - 3 \right) _{\frac{\ell -3}{2}}^2}{2^{\ell -3}} \sum _{r = 0}^{\frac{\ell -1}{2}} \left( -1 \right) ^{\frac{\ell -1}{2} + r} \dfrac{\ell - 2 - 2r}{\nonumber }\\&\quad {\left( 2r \right) ! \left( \ell - 1 - 2r \right) !} \left( 6m+1 \right) ^{2r} \left( 24\ell n - \left( 6m+1 \right) ^2 \right) ^{\frac{\ell -1}{2} - r} \nonumber \\&\equiv \dfrac{16}{2^\ell } \left( \ell -3 \right) _{\frac{\ell -3}{2}}^2 \left( 6m+1 \right) ^{\ell -1} \sum _{r=0}^{\frac{\ell -1}{2}} \dfrac{2r+2}{\left( 2r \right) ! \left( \ell - 1 - 2r \right) !} \nonumber \\&\quad \equiv \dfrac{32}{2^\ell } \left( \ell -3 \right) _{\frac{\ell -3}{2}}^2 \left( 6m+1 \right) ^{\ell -1} \sum _{r=0}^{\frac{\ell -1}{2}} \left( {\begin{array}{c}\ell \!-\!1\\ 2r\end{array}}\right) \dfrac{r+1}{\left( \ell -1 \right) !} \nonumber \\  &\equiv \varrho _\ell \left( 6m+1 \right) ^{\ell -1} \equiv {\left\{ \begin{array}{ll} \varrho _\ell &  m \not \equiv - \overline{6}, \\ 0 &  m \equiv -\overline{6}, \end{array}\right. } \pmod {\ell }, \end{aligned}$$

where

3.3 $$\begin{aligned} \varrho _\ell := \dfrac{32}{2^\ell } \left( \ell -3 \right) _{\frac{\ell -3}{2}}^2 \sum _{r=0}^{\frac{\ell -1}{2}} \left( {\begin{array}{c}\ell -1\\ 2r\end{array}}\right) \dfrac{r+1}{\left( \ell -1 \right) !} \pmod {\ell }. \end{aligned}$$

To compute $\varrho _\ell$, we note that for $M \ge 1$, we have

$$\begin{aligned} \sum _{r=0}^M \left( {\begin{array}{c}2M\\ 2r\end{array}}\right) r = 2^{2M-2} M\ \ \ {\text {and}} \ \ \ \sum _{r=0}^M \left( {\begin{array}{c}2M\\ 2r\end{array}}\right) = 2^{2M-1}. \end{aligned}$$

Therefore, by setting $M = \frac{\ell -1}{2},$ we have

$$\begin{aligned} \sum _{r=0}^{\frac{\ell -1}{2}} \left( {\begin{array}{c}\ell -1\\ 2r\end{array}}\right) (r+1) \equiv \dfrac{\ell -1}{2} 2^{\ell -3} + 2^{\ell -2} = 3\cdot 2^{\ell -4}\pmod {\ell }. \end{aligned}$$

Combining this with (3.3), we obtain

$$\begin{aligned} \varrho _\ell \equiv \dfrac{6 \left( \ell -3 \right) _{\frac{\ell -3}{2}}^2}{\left( \ell -1 \right) !} \pmod {\ell }. \end{aligned}$$

After application of Wilson’s Theorem we see that

$$\begin{aligned} \varrho _\ell \equiv -6 \left( \ell -3 \right) _{\frac{\ell -3}{2}}^2 \pmod {\ell }. \end{aligned}$$

Finally, we note that

$$\begin{aligned} (\ell -3)_{\frac{\ell -3}{2}} \equiv \frac{(-1)^{\frac{\ell -3}{2}}\left( \frac{\ell +1}{2}\right) !}{2} \pmod {\ell }. \end{aligned}$$

Thus

3.4 $$\begin{aligned} \varrho _\ell \equiv -6 \left( \dfrac{(-1)^{\frac{\ell -3}{2}}\left( \frac{\ell +1}{2}\right) !}{2} \right) ^2 \equiv -3\cdot \overline{2} \left( \frac{\ell +1}{2}\right) !^2 \pmod {\ell }. \end{aligned}$$

Therefore, we have by (3.1) and (3.2)

3.5 $$\begin{aligned} \varrho _\ell p\left( \ell n \right)&\equiv \textrm{Tr}_{\ell -1}\left( \ell n \right) + \varrho _\ell \sum _{m \in \mathbb Z\backslash \{0\}} \left( -1 \right) ^{m+1} \left( 6m+1 \right) ^{\ell -1} p\left( \ell n - \omega (m) \right) \nonumber \\  &\equiv \textrm{Tr}_{\ell -1}\left( \ell n \right) + \varrho _\ell \sum _{\begin{array}{c} m \in \mathbb Z\backslash \{0\} \\ 6m \not \equiv -1 \pmod {\ell } \end{array}} \left( -1 \right) ^{m+1} p\left( \ell n - \omega (m) \right) \pmod {\ell }. \end{aligned}$$

Now, substituting $n \mapsto \ell n$ in (2.1) and multiplying by $\varrho _\ell$ on both sides gives

$$\begin{aligned} \varrho _\ell p\left( \ell n \right) \equiv \varrho _\ell \sum _{m \in \mathbb Z\backslash \{0\}} (-1)^{m+1} p\left( \ell n - \omega (m) \right) q^n \pmod {\ell }. \end{aligned}$$

By subtracting (3.5) from this on both sides, we obtain

$$\begin{aligned} 0 \equiv - \textrm{Tr}_{\ell -1}\left( \ell n \right) + \varrho _\ell \sum _{\begin{array}{c} m \in \mathbb Z\\ 6m \equiv -1 \pmod {\ell } \end{array}} \left( -1 \right) ^{m+1} p\left( \ell n - \omega (m) \right) \pmod {\ell }. \end{aligned}$$

Solving for $\textrm{Tr}_{\ell -1}\left( \ell n\right)$ and substituting (3.4) gives the claim. $\square$

We are now ready to prove Theorem 1.1.

### Proof of Theorem 1.1

Proposition 3.1 is equivalent to the generating function congruence

$$\begin{aligned} \mathcal {T}_{\ell }(q) \equiv -3\cdot \overline{2} \left( \frac{\ell +1}{2}\right) !^2 \sum _{n \ge 0} \sum _{\begin{array}{c} m \in \mathbb Z\\ \omega (m) \equiv \delta _\ell \pmod {\ell } \end{array}} (-1)^{m+1} p\left( \ell n - \omega (m) \right) q^{n} \pmod {\ell }, \end{aligned}$$

where we note that $6m \equiv -1 \pmod {\ell }$ is equivalent to $\omega (m) \equiv \delta _\ell \pmod {\ell }$. By taking a convolution product, we see that

$$\begin{aligned} \sum _{n \ge 0} \sum _{\begin{array}{c} m \in \mathbb Z\\ \omega (m) \equiv \delta _\ell \pmod {\ell } \end{array}} (-1)^{m+1} p\left( \ell n - \omega (m) \right) q^{n} \equiv \mathcal {P}_\ell \left( q \right) \theta _\ell (q) \pmod {\ell }, \end{aligned}$$

for some *q*-series

$$\begin{aligned} \theta _\ell (q) := \sum _{s \in \mathbb Z} (-1)^{y_\ell (s)} q^{w_\ell (s)}. \end{aligned}$$

We now turn to the explicit calculation of $\theta _{\ell }(q),$ which then completes the proof. To this end, we observe that the *n*-th Fourier coefficient of $\mathcal {P}_\ell \left( q \right) \theta _\ell (q)$ is

$$\begin{aligned}&\sum _{\begin{array}{c} m \in \mathbb Z\\ \omega (m) \equiv \delta _\ell \pmod {\ell } \end{array}}\!\!\!\!\!\!\! (-1)^{m+1} p\left( \ell n - \omega (m) \right) \\&\quad \equiv \sum _{s \in \mathbb Z} (-1)^{y_\ell (s)} p\left( \ell n - \left( \ell w_\ell (s) + \delta _\ell \right) \right) \pmod {\ell }. \end{aligned}$$

To identify $w_\ell (s)$, we solve $\ell w_\ell (s) + \delta _\ell = \omega (m)$ for $m \equiv -\overline{6} \pmod {\ell }$. Now, define $\alpha _\ell$ by $6\alpha _\ell = \ell m_\ell - 1$ with $m_\ell = \pm 1$ chosen so that $\alpha _\ell = \frac{\ell m_\ell - 1}{6} \in \mathbb Z$. Then by setting $m = \ell s + \alpha _\ell$ in the formula for $\omega (m)$ and simplifying, we see that

$$\begin{aligned} \omega (\ell s + \alpha _\ell ) = \ell \dfrac{3\ell s^2 + 6\alpha _\ell s + s}{2} + \dfrac{3\alpha _\ell ^2 + \alpha _\ell }{2} = \ell \dfrac{3\ell s^2 + \ell m_\ell s}{2} + \delta _\ell . \end{aligned}$$

Thus

$$\begin{aligned} w_\ell (s) = \dfrac{3\ell s^2 + \ell m_\ell s}{2} = {\left\{ \begin{array}{ll} \dfrac{3\ell s^2 + \ell s}{2} &  \text {if } \ell \equiv 1 \pmod {6}, \\ [+0.5cm] \dfrac{3\ell s^2 - \ell s}{2} &  \text {if } \ell \equiv 5 \pmod {6}. \end{array}\right. } \end{aligned}$$

Likewise, by comparing $(-1)^{y_\ell (s)} = (-1)^{m+1}$ if $m = \ell s + \alpha _\ell$ with the same choice of $\alpha _\ell$, we can set $y_\ell (s) = s + \alpha _\ell + 1$. We therefore obtain after some calculation that for $\ell \equiv 1 \pmod {6}$, we have, using (3.1),

$$\begin{aligned}&\theta _\ell (q)=\sum _{s \in \mathbb Z} \left( -1 \right) ^{s+\frac{\ell -1}{6}+1} q^{\frac{3s^2 + s}{2} \ell } = \left( -1 \right) ^{\frac{\ell -1}{6}+1}\\&\quad \sum _{s \in \mathbb Z} \left( -1 \right) ^s q^{\frac{3s^2+s}{2} \ell } = \left( -1 \right) ^{\frac{\ell +5}{6}} \left( q^\ell ;q^\ell \right) _\infty . \end{aligned}$$

Likewise for $\ell \equiv 5 \pmod {6}$ we have, using (3.1),

$$\begin{aligned} \theta _\ell (q)&= \sum _{s \in \mathbb Z} \left( -1 \right) ^{s + \frac{\ell +1}{6} + 1} q^{\frac{3s^2-s}{2} \ell } = \left( -1 \right) ^{\frac{\ell +1}{6}}\\&\quad \sum _{s \in \mathbb Z} \left( -1 \right) ^{s+1} q^{\frac{3s^2-s}{2} \ell } = \left( -1 \right) ^{\frac{\ell -1}{6}} \left( q^\ell ;q^\ell \right) _\infty . \end{aligned}$$

Now note that

$$\begin{aligned} -\left( \frac{-1}{\ell }\right) = {\left\{ \begin{array}{ll} (-1)^{\frac{\ell +5}{6}}& \text {if }\ell \equiv 1\pmod {6},\\ (-1)^{\frac{\ell +1}{6}}& \text {if }\ell \equiv 5\pmod {6}. \end{array}\right. } \end{aligned}$$

We conclude

$$\begin{aligned} c_\ell \equiv -\left( \frac{-1}{\ell }\right) \overline{-3\cdot \overline{2}\left( \dfrac{\ell +1}{2}\right) !^2} \equiv 2\cdot \overline{3} \left( \frac{-1}{\ell }\right) \left( \dfrac{\ell +1}{2}\right) !^{\ell -3} \pmod {\ell }, \end{aligned}$$

which completes the proof. $\square$

## Data Availability

No datasets were generated or analysed during the current study.

## Footnotes

1 Convergence can be concluded from Theorem 1.4 of [4].

2 To avoid confusing notation, we note that $R_{k}(z)$ is denoted $P_{k}(z)$ in [4].

3 We note a small typographical error in [4] (there the $(-1)^{r+1}$ is $(-1)^r$).
